# A review of bioinformatic pipeline frameworks

**DOI:** 10.1093/bib/bbw020

**Published:** 2016-03-24

**Authors:** Jeremy Leipzig

**Affiliations:** Department of Biomedical and Health Informatics, The Children’s Hospital of Philadelphia, 3535 Market Street, Room 1063, Philadelphia, PA, USA

**Keywords:** pipeline, workflow, framework

## Abstract

High-throughput bioinformatic analyses increasingly rely on pipeline frameworks to process sequence and metadata. Modern implementations of these frameworks differ on three key dimensions: using an implicit or explicit syntax, using a configuration, convention or class-based design paradigm and offering a command line or workbench interface. Here I survey and compare the design philosophies of several current pipeline frameworks. I provide practical recommendations based on analysis requirements and the user base.

## Background

Bioinformatic analyses invariably involve shepherding files through a series of transformations, called a pipeline or a workflow. Typically, these transformations are done by third-party executable command line software written for Unix-compatible operating systems. The advent of next-generation sequencing (NGS), in which millions of short DNA sequences are used as the source input for interpreting a range of biological phenomena, has intensified the need for robust pipelines. NGS analyses tend to involve steps such as sequence alignment and genomic annotation that are both time-intensive and parameter-heavy.

**Figure 1:**
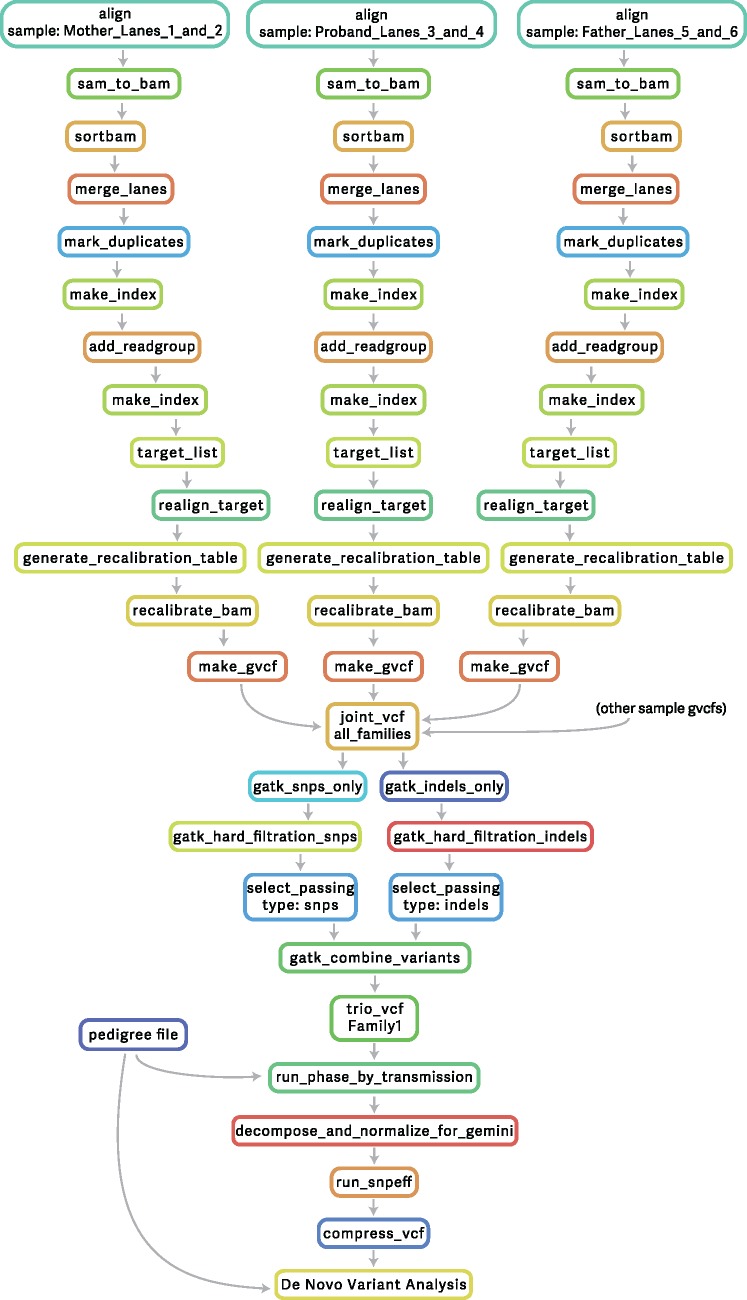
A DAG (Directed Acyclic Graph) depicting a trio analysis pipeline for detecting *de novo* mutations.

A basic exome pipeline delivering called variants from raw sequence could consist of as few as 12 steps, most of which can be run in parallel, but a real analysis will typically involve several additional downstream steps and complex report generation (Figure 1).

Although bioinformatics-specific pipelines such as bcbio-nextgen (https://github.com/chapmanb/bcbio-nextgen) and Omics Pipe [1] offer high-performance automated analysis, they are not frameworks in the sense they are not easily extensible to integrate new user-defined tools. A bioinformatics framework should be able to accommodate production pipelines consisting of both serial and parallel steps, complex dependencies, varied software and data file types, fixed and user-defined parameters and deliverables. Many modern pipeline frameworks offer advanced features, such as displays for visualizing progress in real time, the ability to instantiate containerized tools that can run anywhere, support for performing work on distributed clusters or in the cloud and graphical user interfaces that allow workflows to be built by users without writing code. What distinguishes frameworks from each other is not features but design philosophy. To understand the origins of these frameworks requires closer examination of their predecessors, i.e. scripts and Makefiles.

## Scripts

Scripts, written in Unix shell or other scripting languages such as Perl, can be seen as the most basic form of pipeline framework. Scripting allows variables and conditional logic to be used to build flexible pipelines. However, in terms of ‘robustness’, as defined by Sussman [2], scripts tend to be quite brittle. In particular, scripts lack two key features necessary for the efficient processing of data: support for ‘dependencies’ and *‘*reentrancy’. Dependencies refer to upstream files (or tasks) that downstream transformation steps require as input. When a dependency is updated, associated downstream files should be updated as well. Reentrancy is the ability of a program to continue where it left off if interrupted, obviating the need to restart from the beginning of a process. Pipelines often include steps that fail for any number of reasons such as network or disk issues, file corruption or bugs. A pipeline must be able to recover from the nearest checkpoint rather than overwrite or ‘clobber’ otherwise usable intermediate files. In addition, the introduction of new upstream files, such as samples, in an analysis should not necessitate reprocessing existing samples.

## Make

**Figure 2:**
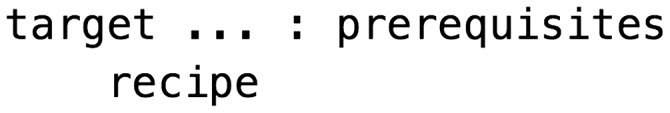
The basic Make rule syntax.

Despite its origin as a compiler build automation tool early in computing history, the Make utility [3] is still successfully used to manage file transformations common to scientific computing pipelines. Make introduced the concept of ‘implicit wildcard rules’, which define available file transformations based on file suffixes (Figure 2).

**Figure 3:**
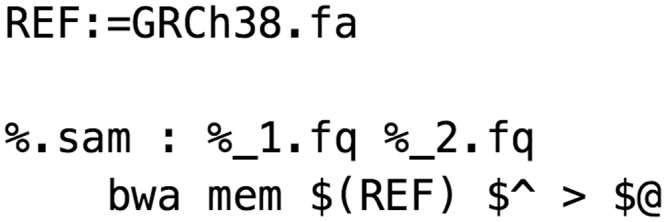
A Make rule for performing a sequence alignment using bwa mem [4]. Two paired fastq (.fq) files are used to produce a SAM alignment file. Symbols are used to represent various pattern matching elements of filenames.

A dependency tree is generated by Make from these rules. When Make is asked to build a downstream file, or ‘target’, file modification datetimes are used to determine whether any of that target’s dependencies are newer than the target or its intermediates. The dependency tree allows Make to infer the steps required to make any target for which a rule chain exists. Make is a ‘domain-specific language’ (DSL)—it provides a convention-based syntax for describing inputs and outputs with special symbols ($<, $@, $.) to represent shortcuts for accessing filename stems, paths and suffixes of both the target and prerequisites (Figure 3).

Because it was never designed for scientific pipelines, Make has several limitations that render it impractical for modern bioinformatic analyses. Make has no built-in support for distributed computing, so dispatching tasks that can be run in parallel on several nodes of a cluster is not easily done within the Make framework. Make’s syntax is restricted to one wildcard per rule and does not allow for lookup tables or other means of associating inputs to outputs other than exact filename stem matching. Although Make allows a low level of shell scripting, more sophisticated logic is difficult to implement.

## Modern pipeline frameworks

In recent years, a number of new pipeline frameworks have been developed to address Make’s limitations in syntax, monitoring and parallel processing as well as offer new features relevant to bioinformatics and reproducible research, such as visualization, version tracking and summary reports (Table 1).

**Table 1:** A classification of modern pipeline frameworks

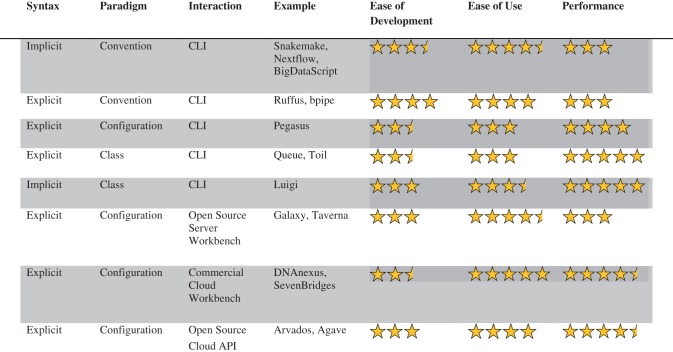										

*Note*. Ease of development refers to the effort required to compose workflows and also wrap new tools, such as custom or publicly available scripts and executables. Ease of use refers to the effort required to use existing pipelines to process new data, such as samples, and also the ease of sharing pipelines in a collaborative fashion. Performance refers to the efficiency of the framework in executing a pipeline, in terms of both parallelization and scalability. More stars connotes ‘easier’ or ‘faster’.

## Implicit convention frameworks

Implicit frameworks preserve the implicit wildcard idioms introduced by Make while extending its capabilities, usually by leveraging full-featured scripting languages such as Python to implement logic both inside and outside of rules.

**Figure 4:**
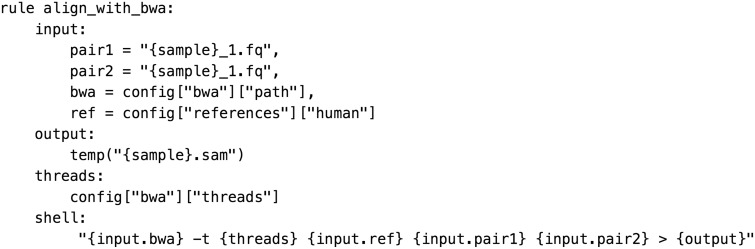
A Snakemake rule for performing a sequence alignment. This example uses a global configuration dictionary that allows parameters to be specified in JSON or YAML-formatted configuration files or in the command line.

Snakemake [5] builds on the implicit or wildcard-based logic of Make while extending its capabilities by allowing Python to be interspersed through the pipeline in conjunction with a DSL. Some implicit frameworks, such as Nextflow (http://nextflow.io), provide tools to abstract and manage filenaming into global variables to reduce ambiguity. BigDataScript [6] is a stand-alone DSL that offers its own language-independent syntax for implementing pipeline logic (Figure 4).

**Figure 5:**
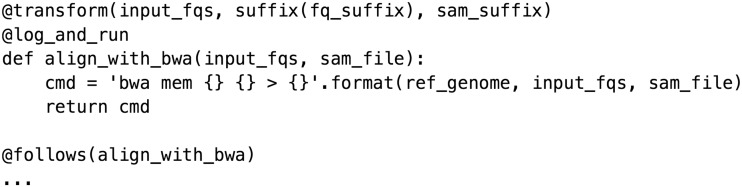
Tasks in Ruffus explicitly depend on other tasks, not file targets.

## Explicit frameworks

Implicit frameworks demand the user define rules or recipes for performing file transformations separately from target(s). Although this approach is logical from the standpoint of defining individual rules, users typically have a preconceived idea of the order of operations. Implicit frameworks force users to think more carefully about filenames rather than about the process. In response, some frameworks such as Ruffus [7] and bpipe [8] use an explicit paradigm, as used in scripts, in which the rule topology is defined by the user, the order is fixed and tasks simply refer to each other rather than using a target naming scheme (Figure 5).

## Configuration frameworks

**Figure 6:**
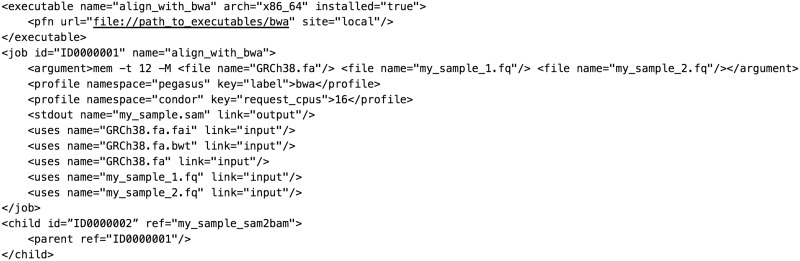
A Pegasus DAX (Directed Acyclic Graph in XML). A subsequent step to alignment has been included to show that a Pegasus task relies on explicit job IDs to identify its antecedents rather than a filename pattern to identify its dependencies. Pegasus has no built-in system of variable injection, but includes APIs to produce DAX files.

Many pipeline frameworks dispense with inline scripting code and instead use a configuration-based, rather than convention-based, means of describing tasks. Pegasus [9] is a National Science Foundation (NSF)-funded workflow system originally designed for the physical sciences. Like all configuration-based frameworks, Pegasus is explicit—it does not implicitly infer how to produce targets but instead requires a fixed XML file that describes individual job run instances and their dependencies (Figure 6).

## Class-based frameworks

Some high-performance workflow languages are implemented in a class-based pure language manner. Although these may resemble DSL-based frameworks superficially, class-based implementations are often closely bound to an existing code library rather than various executables. Class-based pipelines often contain many thousands of lines of code implementing domain logic. Genome Analysis Toolkit (GATK) [10] is a large Java library for variant analysis, and Queue is a GATK-integrated Scala framework that provides abstract classes for implementing pipelines. Luigi (https://github.com/spotify/luigi) and Toil (https://github.com/bd2kgenomics/toil) are pure-Python frameworks that are not bound to any bioinformatics codebase, but offer explicit Application Programming Interfaces (APIs) for defining task dependencies from within task methods. Luigi places particular emphasis on scheduled execution, monitoring, visualization and the implicit dependency resolution of tasks. Toil offers a strong focus on cloud execution.

Many existing implementations of bioinformatics software tend to work with large ‘monolithic’ disk-based files, which impedes the ability of work tasks to be efficiently farmed out to individual cores or nodes in a cluster, or to ephemeral machine instances in the cloud. Efforts such as Big Data Genomics (http://bdgenomics.org) aim to make common data formats ‘splittable’ for use with Hadoop and Spark-based scalable distributed computing frameworks. These efforts will likely also require the use of new or existing class-based pipelines for tasks to be tightly coupled to individual data structures within the library, allowing a high level of granularity in terms of the concurrent processing of data.

## Server workbenches

**Figure 7:**
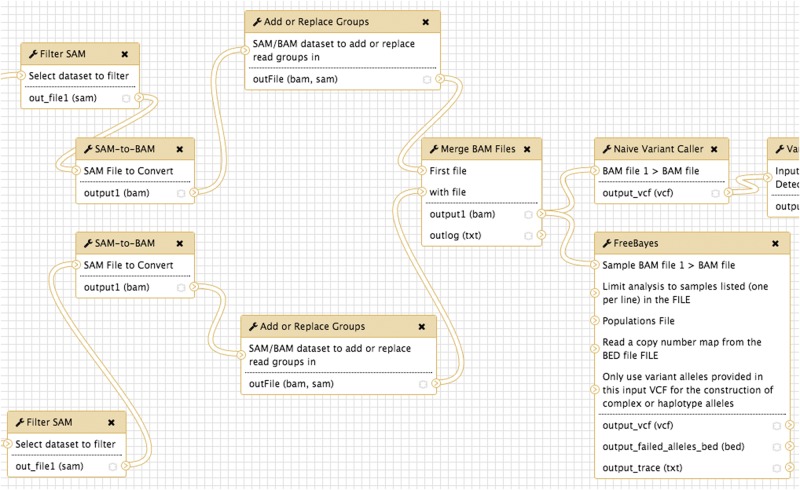
The Galaxy Workflow Editor allows users to link inputs, outputs and tools on a graphical canvas.

Unlike the command line-based pipeline frameworks reviewed previously, workbenches allow end-users, typically scientists, to design analyses by linking preconfigured modular tools together, typically using a drag-and-drop graphical interface. Because they require exacting specifications of inputs and outputs, workbenches are intrinsically a subset of configuration-based pipelines. The most popular bioinformatics server workbenches are Galaxy [11] and Taverna [12]. Galaxy serves as a Web-based interface for command line tools, whereas Taverna offers stand-alone clients and allows pipelines to access tools distributed across the Internet. Both allow users to share workflows and are intended for local installations (Figure 7).

For existing tools that have an existing component plug-in, using Taverna is an easy solution for end-users. Creating a new plug-in requires an in-depth knowledge of the XML-based API and exact specifications of acceptable input filetypes, parameter values, resource management and exception behavior. The onus is entirely on the developer to provide a means for new tools to exist in the Taverna ecosystem. Adding a new executable to Galaxy often requires only 20 lines of configuration code, but Galaxy wrappers can be less robust than those in Taverna, which requires slightly more familiarity with each tool on the part of end-users to implement.

## Cloud workbenches and APIs

Cloud computing, defined here as the on-demand rent of virtualized computing infrastructure from remote managed data centers, offers an attractive scalable option for collaborative multi-institutional research in terms of ‘bringing the tools to the data’. Although subscription and compute costs are decreasing, the speed of file transfer over the Internet to the cloud remains an issue for these platforms. While all of the aforementioned pipelines can be installed on cloud infrastructure [13], cloud workbenches offer a layer of abstraction that simplifies the complex process of provisioning servers.

Commercial workbenches, such as DNAnexus (http://dnanexus.com), SevenBridges (http://sbgenomics.com) and Illumina’s BaseSpace (http://basespace.illumina.com), leverage the scalability of cloud computing to offer high performance while offering development and user experiences comparable with local server-based open source workbenches. These providers also support APIs that allow users to launch automated large batch analyses without using a Web interface.

Next-generation cloud-based open source workbenches, such as Curoverse’s Arvados (https://curoverse.com) and the iPlant Collaborative’s Agave [14], largely dispense with the Web GUI as a primary design tool and instead are built from the ground up as APIs designed to enable the migration of local analyses to the cloud for collaborative research.

## Future trends

**Figure 8:**
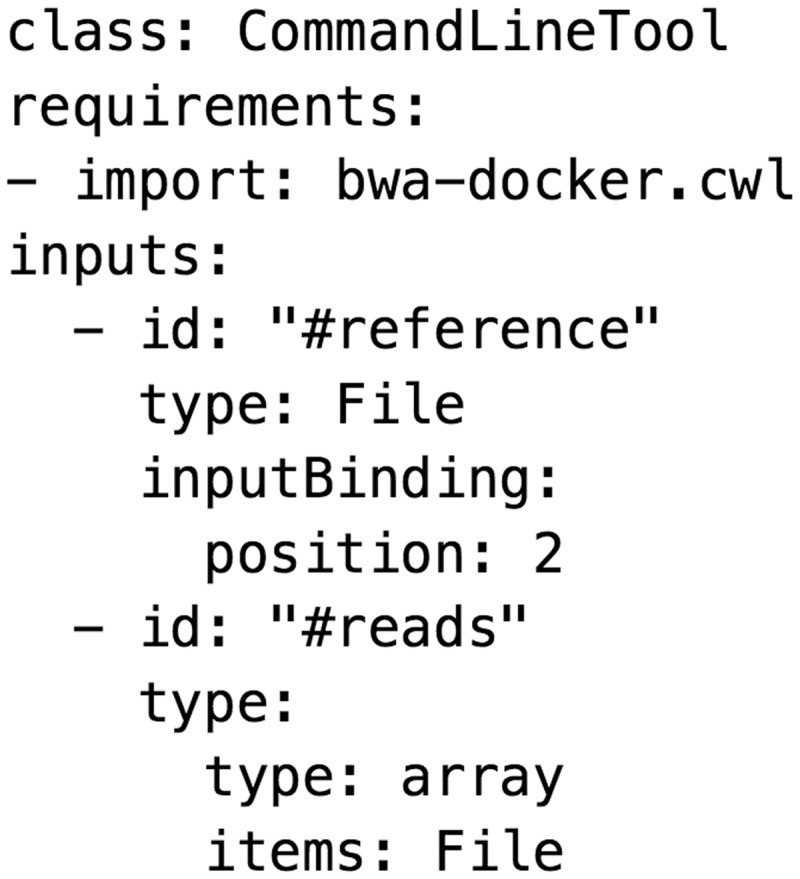
A snippet of the common workflow language describing the bwa mem alignment program.

The need for a consistent means of distributing popular tools among so many frameworks is driving an effort to standardize workflow description languages. The Common Workflow Language Specification (CWL; https://github.com/common-workflow-language) describes a shared platform for developing new tool descriptors, which has particular utility in supporting cloud-enabled workbenches and plug-ins. Among the frameworks reviewed here, Taverna, Galaxy, Toil, Arvados and SevenBridges have already made significant progress toward supporting the CWL. Another promising trend is the containerization of bioinformatic tools using Docker, lightweight virtualization software, which will enable frameworks to easily accommodate tools with complex software dependencies (Figure 8).

## Choosing a pipeline framework

Although there is no formal study of bioinformatics pipeline users specifically, a previous survey (https://github.com/michaelbarton/bioinformatics-career-survey) suggests the audience for bioinformatics development is evenly mixed between those with biological and computer science backgrounds, and large and small institutions. The choice of framework should be informed both by the demands of developing the pipeline and the requirements of those using it, even if the developers and end-users are the same people. The use of pipeline frameworks is intimately tied to reproducible computational research [15], as *ad hoc* analyses are not likely to be implemented in a pipeline. Reusable pipelines that can be run in the cloud are often preferable in terms of reproducible research and the type of collaborative ‘big science’ popular in modern sequencing studies.

Choosing between an implicit or explicit syntax is largely a question of personal preference. To developers unfamiliar with Make rule syntax, arranging a series of implicit wildcard rules and trusting the engine to infer a dependency tree can seem unintuitive, but this idiomatic style offers a high level of convenience for integrating executable tools.

Convention-based frameworks tend to encourage a high level of internal business logic. They also allow polished deliverables (Web sites, PDF reports) to be easily generated from the underlying data. At the same time, pipelines that ‘think on their feet’ would seem inherently less reproducible when compared with configuration-based pipelines that demand a paper trail, but often the latter simply forces developers to write dynamic tools to generate static configurations. Because they are ‘set in stone’, configuration-based pipelines often enable cluster schedulers to consume an entire work plan in entirety instead of receiving tasks in a piecemeal fashion, allowing the scheduler to better anticipate load and allocate both memory and compute resources.

Workbenches and class-based frameworks can be considered heavyweight. There are costs in terms of flexibility and ease of development associated with making a pipeline accessible or fast. Integrating new tools into workbenches clearly increases their audience, but, ironically, the developers who are most capable of developing plug-ins for workbenches are the least likely to use them. Class-based frameworks offer a high level of performance, but like workbenches, require highly skilled developers to build and maintain, and performance improvements are not guaranteed to justify additional development time. The transition to high-performance computing (HPC) frameworks will likely favor class-based pipeline frameworks in the future, although this will severely limit the number of developers who will be able to contribute to these pipelines, owing to their inherent complexity of HPC development compared with DSLs. A recent survey of institutions using bioinformatics pipelines [16] found that virtually every participant anticipated further use of HPC-enabled pipelines in the future and had struggled with issues of reproducibility and data provenance. These issues require intense attention to implementing highly customized solutions that do not lend themselves to lightweight pipelines.

For those laboratories that neither serve a large number of pure biologists who demand a workbench interface nor require the high level of performance that class-based pipelines offer, a clear choice is not so obvious. One heuristic for choosing a framework to consider is ‘return on investment’. Laboratories that conduct large-scale, highly repetitive research requiring a high degree of data provenance and versioning may benefit from configuration-based pipelines. Laboratories doing exploratory proofs-of-concept would see little reason to use more heavyweight frameworks—explicit DSL-based pipelines are adequate.

Finally, most laboratories, especially those without access to internal HPC resources, should consider cloud-based workbenches and APIs. These offer many of the features of server-based frameworks, with the added bonus of unlimited scalability and collaborative research opportunities, albeit incurring direct costs.

Although this review is not intended to be an exhaustive list of pipeline frameworks, such lists do exist (e.g. https://github.com/pditommaso/awesome-pipeline). For laboratories relying solely on scripts, the choice of a framework, especially one to accommodate new custom tools, may seem overwhelming and irreversible, but all frameworks use the parameterization of inputs, outputs and tool descriptors. Once a script-based pipeline is implemented in one framework, transitioning to a different one is relatively simple should priorities change.

**Key Points**

- Key pipeline concepts of dependency and reentrancy were introduced by Make.
- Pipelines are best distinguished not by features but by design philosophy.
- Modern bioinformatic frameworks use a convention, configuration or class-based design paradigm and use an explicit or implicit syntax.
- Workbenches and class-based frameworks offer ease of use and performance, respectively, but require additional investment in time and expertise to integrate new tools.
- Cloud-based platforms offer scalability and collaborative research advantages.
- Developers choosing a pipeline framework should consider the return on investment when considering more heavyweight options.

## Notes

**Jeremy Leipzig** is a bioinformatics software developer at The Children’s Hospital of Philadelphia. He received his MS in Computer Science from North Carolina State University. He is pursuing a PhD in Information Studies at Drexel University.
